# Preparation of Phenolic Epoxy-Based Electronic Packaging Materials with High Thermal Conductivity by Creating an Interfacial Heat Conduction Network

**DOI:** 10.3390/polym17111507

**Published:** 2025-05-28

**Authors:** Minghao Ye, Jing Jiang, Lin Zhao, Hongyu Zhu, Junjie Wang, Zicai Sun, Dewei Zhang, Ming Li, Yagang Zhang

**Affiliations:** 1 School of Materials and Energy, University of Electronic Science and Technology of China, Chengdu 611731, China; 202221210139@std.uestc.edu.cn (M.Y.); 202321030310@std.uestc.edu.cn (J.J.); zhaolin316@uestc.edu.cn (L.Z.); zhy@uestc.edu.cn (H.Z.); 2 Department of Chemistry, School of Science, Xihua University, Chengdu 610039, China; 3 Dongguan Yimei Material Technology Co., Ltd., Dongguan 523000, China; 4 Sichuan Xinyi Electronic Materials Co., Ltd., Chengdu 611731, China; zhang-dewei@163.com (D.Z.); liming@xinyi-emc.com (M.L.)

**Keywords:** electronic packaging materials, high thermal conductivity, epoxy molding compounds, interfacial conduction networks

## Abstract

As one of the most widely used packaging materials, epoxy composite (EP) offers excellent insulation properties; however, its intrinsic low thermal conductivity (TC) limits its application in high-frequency and high-power devices. To enhance the TC of EP, six highly thermally conductive inorganic fillers, namely, Al_2_O_3_, MgO, ZnO, Si_3_N_4_, h-BN, and AlN, were incorporated into the EP matrix at varying contents (60–90 wt.%). The resulting epoxy molding compounds (EMCs) demonstrated significant improvement in thermal conductivity coefficient (λ) at high filler contents (90 wt.%), ranging from 0.67 W m^−1^ K^−1^ to 1.19 W m^−1^ K^−1^, compared to the pristine epoxy composite preform (ECP, 0.36 W m^−1^ K^−1^). However, it was found that the interfacial thermal resistance (ITR) between EP and filler materials is a major hindrance restricting TC improvement. In order to address this challenge, graphene nanosheets (GNSs) and carbon nanotubes (CNTs) were introduced as additives to reduce the ITR. The experimental results indicated that CNTs were effective in enhancing the TC, with the optimized EMC achieving a λ value of 1.14 W m^−1^ K^−1^ using 60 wt.% Si_3_N_4_ + 2 wt.% CNTs. Through the introduction of a small amount of CNT (2 wt.%), the inorganic filler content was significantly reduced from 90 wt.% to 60 wt.% while still maintaining high thermal conductivity (1.14 W m^−1^ K^−1^). We propose that the addition of CNTs helps in the construction of a partial heat conduction network within the EP matrix, thereby facilitating interfacial heat transfer.

## 1. Introduction

The electronic packaging process of enclosing or encapsulating chips in protective materials is crucial for ensuring the physical and electrical protection, thermal management, and mechanical mounting of electronic devices. Over the past few decades, advancements in communication and semiconductor technologies have driven the evolution of electronic devices toward higher frequencies, improved performance, and increased power consumption, resulting in greater heat generation during operation. It is well known that overheating or thermal runaway in electronic devices poses a serious threat to their safety [1,2], and can even cause device combustion or explosion. Therefore, the development of packaging materials with high thermal conductivity (TC) is essential for maintaining the stability, safety, and performance of electronic equipment.

Epoxy composites (EPs) are widely used in electronic packaging materials due to their excellent insulating properties, high temperature resistance, good chemical stability, and excellent mechanical strength [3]. However, the inherently low thermal conductivity coefficient (λ) of EP hinders (0.2 W m^−1^ K^−1^) effective heat dissipation in high-performance electronic devices [4,5,6]. Therefore, enhancing the λ value of EPs has become a hot topic in the field of electronic packaging. Among the various methods used to address this limitation, incorporating inorganic fillers with a high λ value into an EP matrix to form epoxy molding compounds (EMCs) is one of the most commonly used strategies [7,8]. Aluminum oxide (Al_2_O_3_), boron nitride (BN), aluminum nitride (AlN), and other ceramic powders have proven effective in improving the TC of EP [9,10,11,12]. For example, Sanchez et al. incorporated 80 wt.% Al_2_O_3_/BN hybrid filler into the EP matrix, obtaining EMC with a λ value achieving 1.72 W m^−1^ K^−1^ [13]; Wang et al. prepared EMC with a λ value of 1.64 W m^−1^ K^−1^ by using 1.5 wt.% silicon nitride (Si_3_N_4_) nanowires and 70 wt.% AlN as the filler [14]. Isarn et al. synthesized EMC with a λ value of 1.21 W m^−1^ K^−1^ by using 75 wt.% AlN as the filler [15].

Typically, improving the TC of EP often necessitates the relatively high filler loading of thermally conductive inorganic materials. High filler contents can provide sufficient contact between filler particles, thus reducing the interfacial thermal resistance (ITR) between the EP and filler materials, as well as forming heat conduction pathways in the EP matrix for heat dissipation [16]. Following this principle, considerable efforts have been devoted to reducing ITR and creating heat conduction pathways, including optimizing filler loading and dispersion, enhancing the interfacial interactions between polymer and fillers, designing and synthesizing polymers and filler materials with specialized structures and morphologies, etc. [17,18,19]. However, a high content of fillers in an EP matrix still causes problems, such as decreased mechanical properties, poor processing performance, and high costs [20]. Therefore, developing EP packaging materials with relatively low filler content while maintaining a high TC is still challenging in this field.

Recently, carbon nanotubes (CNTs) have garnered significant attention owing to their remarkable electrical, thermal, and mechanical properties [21,22]. Numerous studies have demonstrated that incorporating CNTs into the EP matrix can significantly enhance the TC of the prepared EMCs. The superior thermal performance of EMCs can be attributed to the elongated tubular structure and high λ value of CNTs, which enables the formation of efficient heat transfer networks within the polymer matrix [23,24]. However, excessive usage of CNTs may negatively affect the insulating properties of EMCs, potentially causing a chip short circuit.

**Figure 1:**
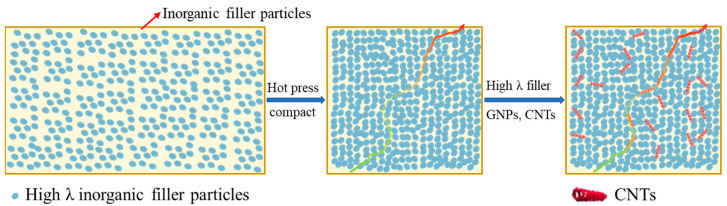
Preparation of phenolic epoxy-based electronic packaging materials with high thermal conductivity by adding inorganic filler and by creating interfacial heat conduction networks with the CNTs.

To overcome the disadvantages associated with incorporating high contents of fillers and CNTs, this study incorporated CNTs as additives to epoxy composite preform (ECP) material to reduce filler loading while achieving EMCs with high TC. Once incorporated into the matrix, the high λ value of CNTs facilitated rapid heat transfer within the matrix, thereby improving the TC of EMCs. As shown in Figure 1, the elongated tubular structure of CNTs could also establish direct contact with multiple inorganic fillers at the nanoscale, forming partial heat conduction pathways and networks that reduce the ITR of the EMCs.

As a result, the λ value of the prepared EMC containing 60 wt.% Si_3_N_4_ and 2 wt.% CNTs reached a λ value of 1.14 W m^−1^ K^−1^, which is comparable to that of the EMC with 90 wt.% Al_2_O_3_. This approach advances the use of traditional methods that rely on high filler loadings of inorganic materials to construct heat conduction networks through particle-to-particle contact. Instead, a localized heat conduction network was established using CNTs, thus enabling the fabrication of highly thermally conductive EMCs with relatively low inorganic filler content. The newly prepared EMCs with CNT additives exhibited an improvement in λ value of up to 216% compared to the pristine ECP.

## 2. Experimental Section

### 2.1. Materials

The chemicals used in this study are listed in Table 1, and all chemicals were used without further purification.

### 2.2. Preparation of EMCs

The ECP was first prepared with the assistance of Sichuan Xinyi Electronics Co., Ltd., Chengdu, China, Following the formulation outlined in Table 2, the raw materials were sequentially mixed, melted, and kneaded together using a twin-screw extruder (CT30/15, Jiangsu Aurode Machinery Co., Ltd., Yangzhou, China) for 5 min. After extrusion, crushing, and compression, the resulting product was further milled and pre-cured at 130 °C for 2 h, followed by post-curing at 180 °C for 5 h to produce the ECP.

To begin the process, 100 g of ECP was used as the matrix, to which different mass percentages and types of fillers were added, and initial mixing was performed by mechanical stirring. The resulting mixture was extruded in a twin-screw extruder at a speed of 50 rpm and at an extrusion temperature of 90 °C. The temperature of each section of the extruder was set to 45 °C in the feed section and 90 °C in both the compression and melting sections. After pulverization and hot-pressing using a vulcanizing machine at 180 °C, EMC samples were obtained for further research. The manufacturing process comprised two sequential phases: (1) mixing, employing twin-screw extrusion with a fixed L/D ratio, synchronized at 50 rpm screw speed and 90 °C barrel temperature (with the feed zone independently regulated at 45 °C); (2) forming, involving a 180 °C/2 h curing cycle within a Φ40 mm circular stainless steel mold cavity to produce specimens with standardized 4 mm cross-sectional thickness.

### 2.3. Characterization of EMCs

#### 2.3.1. Field Emission Scanning Electron Microscopy (FESEM)

The surface topography of the EMC samples was examined using an Ultim Max40 field emission scanning electron microscope (Oxford Instruments, Abingdon, UK). The crushed EMC was sputtered under a vacuum for 50 s and then observed at an accelerating voltage of 10 kV. The EDS detector model used in this study was the Ultim Max40 (Oxford Instruments, Abingdon, UK).

#### 2.3.2. Fourier-Transform Infrared (FT-IR) Spectroscopy

FT-IR measurements were performed in attenuated total reflection (ATR) mode using an Invenior spectrometer (Bruker Co., Billerica, MA, USA). The 20 mg epoxy molding compound (EMC) sample was placed in the crystal sample chamber, and the optical coupling between the sample and the crystal interface was achieved through a diamond anvil. The wavenumber range was 400–4000 cm^−1^ (covering the mid-infrared characteristic absorption region), the resolution was 4 cm^−1^, and a total of 32 scans were conducted to improve the signal-to-noise ratio.

#### 2.3.3. TCT-S2 Thermal Conductivity Test

**Figure 2:**
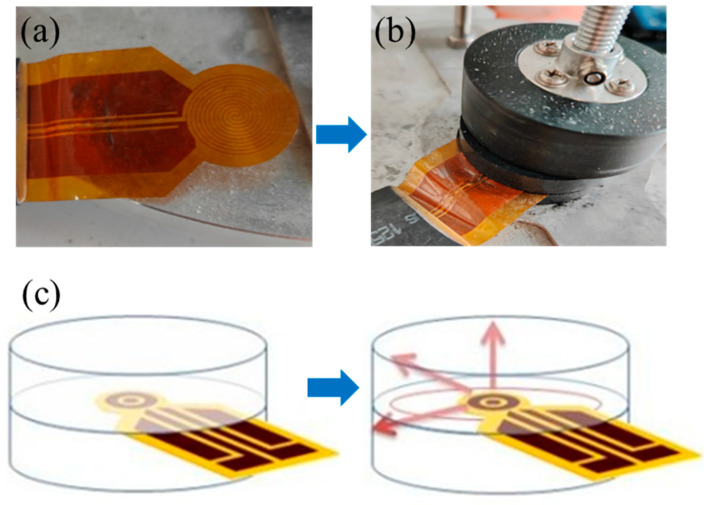
(**a**) Planar probe with a double helix structure. (**b**) Measurement process. (**c**) Mechanism of the TCT-S2 test to determine λ values.

The TCT-S2 technique is a testing method developed based on transient planar heat source (TPS) technology to evaluate the λ values of EMCs. A planar probe with a double helix structure (Figure 2a) was employed as both the heat source and the temperature sensor. During the measurement, the planar probe was placed between two pieces of EMC materials, as shown in Figure 2b, and a short heat pulse was applied to the probe to generate heat within the material. The measurement process typically uses a Wheatstone bridge circuit to detect the small changes in voltage caused by the temperature changes (Figure 2c). Therefore, the voltage can be directly related to the thermal response of the materials. The acquired data were analyzed and processed via computer software (AVIC Era Instruments thermal conductivity analysis and testing software, Version 24.03.291), ultimately yielding the λ values of the EMC sample. The measurements were operated in accordance with the ISO 22007-2:2022 standard [25].

#### 2.3.4. Water Evaporation Experiments

Water evaporation experiments were conducted to make a direct and visual comparison of the TC values between different materials. Specifically, the compressed bulk EMC sample was placed on a heating plate set at 150 °C. Afterward, 500 μL of deionized water was carefully dropped on the center of the composite surface, and an initial picture was taken immediately. Subsequent images were captured at 3-minute intervals over a total duration of 15 min. By observing and comparing the state of the water droplets, the TC of different materials could be directly compared and assessed. (Supplementary statement: “The water evaporation experiment is only a qualitative verification of thermal performance, and quantitative data is subject to the results of the standard thermal conductivity test”).

## 3. Results and Discussion

### 3.1. λ Values of EMCs with Different Inorganic Fillers

Based on previous studies, six typical and widely used inorganic fillers with high λ values were selected for further research, and their λ values are listed in Table 3. (All thermal conductivity coefficients (λ values) were measured using the transient plane heat source method (TPS) described in Section 2.3.3).

These six inorganic fillers were incorporated into ECP at designed mass ratios (Table 4) to synthesize a series of EMCs; their corresponding λ values are presented in Table 4. In general, the λ values of all the prepared EMCs were enhanced (Table 4, Entry 1 vs. Entries 2–10), with the highest λ value achieving 1.19 W m^−1^ K^−1^ by using 90 wt.% Al_2_O_3_ as filler.

Furthermore, replacing the fillers of Al_2_O_3_ with AlN or Si_3_N_4_ at the same filler content (90 wt.%) resulted in a decrease in TC (Table 4, Entry 2 vs. Entries 3 and 4). Notably, the λ values of the EMCs synthesized with Al_2_O_3_ or AlN as the filler displayed a marked decrease in response to a decrease in filler content (Table 4, Entries 2 and 3 vs. Entries 5 and 6), while the λ value of EMCs using Si_3_N_4_ as filler exhibited an improvement from 0.67 W m^−1^ K^−1^ to 0.86 W m^−1^ K^−1^ when the filler content decreased from 90 wt.% to 60 wt.% (Table 4, Entry 4 vs. Entry 7). The lower λ values with higher filler contents can primarily be attributed to the agglomeration of Si_3_N_4_ filler, which disrupts the formation of efficient thermal conductive pathways [32,33]. In addition, EMCs containing other ceramic fillers, such as magnesium oxide (MgO), zinc oxide (ZnO), and h-BN at 60 wt.% content did not exhibit superior TC values compared to EMCs with Si_3_N_4_ filler (Entries 8–10 vs. Entry 7). Therefore, while EMCs incorporating Al_2_O_3_ filler exhibited the highest λ value of 1.19 W m^−1^ K^−1^ at high filler contents (90 wt.%), EMCs containing Si_3_N_4_ filler achieved the highest λ value of 0.86 W m^−1^ K^−1^ at a comparatively lower filler content (60 wt.%).

### 3.2. ITR Between the EP Matrix and Inorganic Filler

Compared to the intrinsic λ values of inorganic fillers (10–380 W m^−1^ K^−1^, Table 1), the λ values of the EMCs incorporating these fillers remained relatively low (0.44–1.19 W m^−1^ K^−1^, Table 4). The limitation to TC enhancement by incorporating inorganic fillers can be attributed to two main factors:

(I). The morphology and structure of inorganic fillers have a significant impact on their λ values. The theoretical λ values of the added inorganic fillers are calculated or measured by assuming a state of perfect crystalline structures, while the actual inorganic fillers utilized in this study were amorphous particles, which have λ values lower than those of perfect crystals.
(II). The ITR between the EP matrix and inorganic fillers significantly influences the overall TC of the prepared EMCs.

Although the precise correlation between interfacial structure, including interfacial interactions, ITR, and the λ values of EMCs remains unclear [34,35,36], an approximate relationship between λ values and the ITR was still established through numerical simulation and the use of an effective medium model, as presented by Equations (1) and (2) [37]:

(1) $$K=K_{m}\frac{3+V_{f}\left(\beta_{⊥}+\beta_{∥}\right)}{3-V_{f}\beta_{⊥}}$$

**Figure 3:**
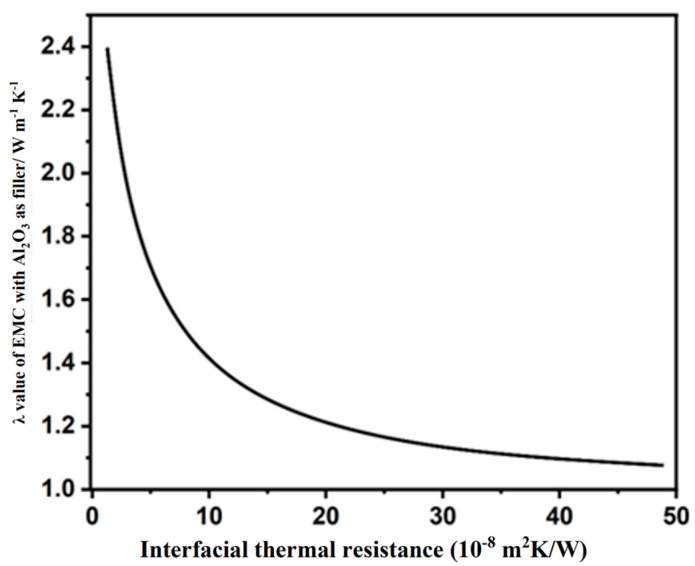
Effect of ITR on the λ value of the EMC.

With:

(2) $$\beta_{⊥}=\frac{2(d\left(K_{f}-K_{m}\right)-2R_{C1}K_{f}K_{m})}{d\left(K_{f}+K_{m}\right)+2R_{C1}K_{f}K_{m}},\;\;\beta_{∥}=\frac{L\left(K_{f}-K_{m}\right)-2R_{C1}K_{f}K_{m}}{LK_{m}+2R_{C1}K_{f}K_{m}}$$

where K, $K_{m}$, and $K_{f}$ represent the λ values of the EMCs, ECP, and fillers, respectively. $V_{f}$ stands for the volume loading of fillers. $R_{C1}$ represents the interfacial resistance. L and d represent the length and diameter of the fillers, respectively. In order to intuitively analyze the influence of the particle size of the filler on ITR in the experiment, the relevant parameters of ECP and Al_2_O_3_ fillers were substituted into Equations (1) and (2); the correlation between the EMC λ value and ITR is shown in Figure 3.

**Figure 4:**
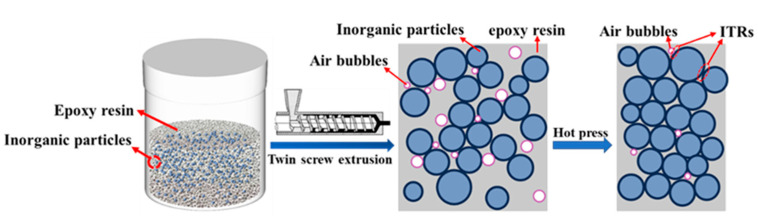
Reduction of the ITR in ECP using a twin-screw extruder.

In general, the λ values of the EMC demonstrate a rapid decrease when the ITR is below 1 × 10^−7^ m^2^ K W^−1^, whereas the decrease in λ values becomes significantly less pronounced as the ITR exceeds 1 × 10^−7^ m^2^ K W^−1^. Calculations indicate that the ITR is negatively correlated with the λ values of the EMC when $R_{K}$ < 1 × 10^−7^ m^2^ K W^−1^. Therefore, a reduction in ITR is crucial for enhancing λ values. Reducing interfacial thermal resistance (ITR) was achieved by enhancing the interfacial contact between fillers and the epoxy resin (EP) matrix, along with minimized bubble entrapment. To achieve this, a twin-screw extruder operating at 50 rpm and 90 °C was employed for filler/EP blending. Figure 4 illustrates the optimized processing parameters and the mixing mechanism.

A twin-screw extruder can achieve the continuous and efficient curing of epoxy resin composites, and can accurately control the mixing and reaction process through multi-stage temperature control and strong shear force to improve the uniformity and performance of the material. By adjusting the speed and temperature of the extruder, the inorganic particles were uniformly dispersed throughout the matrix (vide infra). This uniform dispersion helps establish thermally conductive pathways through enhanced filler-to-filler connectivity, thereby reducing the ITR [38,39]. Additionally, the extruded composites were subjected to compression using a plate vulcanizer, which expelled the air bubbles between the ECR material and the filler. This process facilitated direct contact between the fillers, further reducing the ITR.

### 3.3. λ Values of EMCs with Fillers and CNTS

#### Characterization of the Molecular Chain Structure

**Figure 5:**
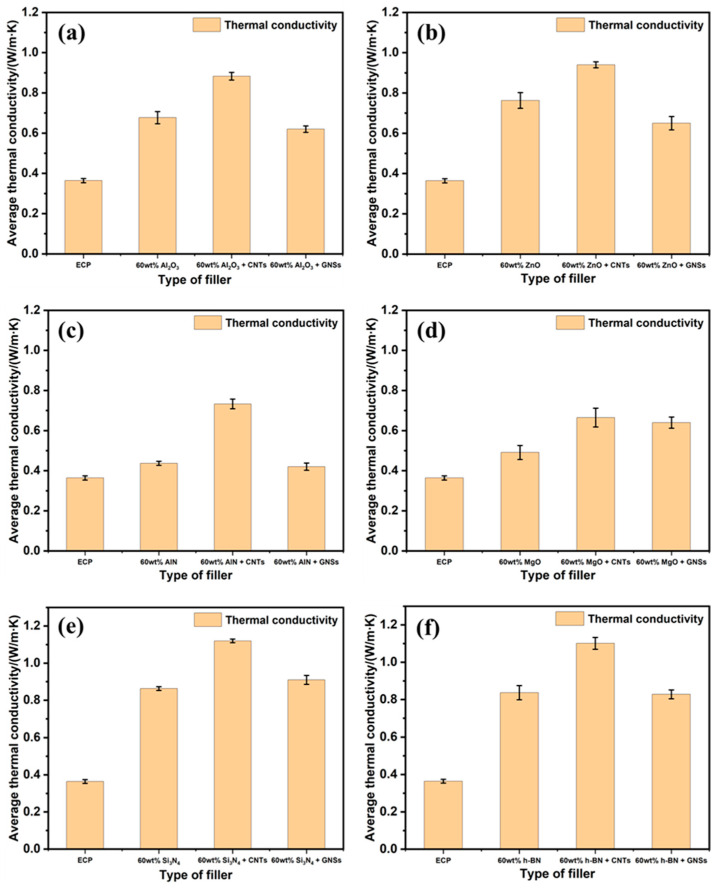
(**a**–**f**) λ values of EMCs incorporating different fillers and additives.

Generally, the compression of polymer campsites with inorganic fillers to reduce ITR often necessitates a high content of filler to ensure that there is sufficient contact between the filler particles to form heat conduction pathways. However, an excessive addition of thermally conductive fillers will inevitably cause problems such as decreased mechanical properties in the packaging material, poor processing performance, and costliness [40]. To circumvent these disadvantages, the incorporation of thermally conductive additives offers another effective pathway to reduce the ITR. Moreover, the use of additives enables a reduction in filler content, which provides the added benefits of good mechanical properties and low costs. Therefore, by fixing the content of the inorganic filler at 60 wt.%, two representative additives, i.e., GNSs and CNTs, were incorporated into the EP matrix, respectively. The EMCs (60 wt.% of inorganic fillers + 2 wt.% of GNSs or CNTs) were prepared with a twin-screw extruder and their λ values are presented in Figure 5 (for details, please refer to Table S1 in the Supplementary Materials).

The results demonstrated that CNTs can significantly improve the λ values of EMCs to 0.68–1.14 W m^−1^ K^−1^ (Figure 5a–f), among which values, EMC incorporated with 60 wt.% Si_3_N_4_ + 2 wt.% CNTs achieved the highest λ value of 1.14 W m^−1^ K^−1^. In addition, EMC incorporated with 60 wt.% h-BN + 2 wt.% CNTs also exhibited a high λ value of 1.10 W m^−1^ K^−1^, indicating the versatility of this method. Notably, a remarkable improvement in λ value reaching 76% was obtained by adding 2 wt.% CNTs into EMC with 60 wt.% AlN, showing the significant impact of CNT additives on TC. In contrast, CNSs only slightly enhance the λ values of EMCs, and in some cases, they even reduce the λ values of EMCs (Figure 5a–f). For example, adding 2 wt.% of GNSs to EMCs containing 60 wt.% MgO or Si_3_N_4_ resulted in slight increases in the λ values of 0.15 and 0.05 W m^−1^ K^−1^, respectively (Figure 5d,e). The λ value of EMCs with 60 wt.% h-BN remained almost unchanged with the addition of 2 wt.% of GNSs. However, GNSs even negatively impacted the TC of EMC with 60 wt.% Al_2_O_3_, AlN, and ZnO, exhibiting a decrease in λ value by 7.4–22.6% (Figure 5a–c). The λ value data after adding CNTs/GNSs (Figure 5a–f) is also based on the standardized testing process described in Section 2.3.3.

**Figure 6:**
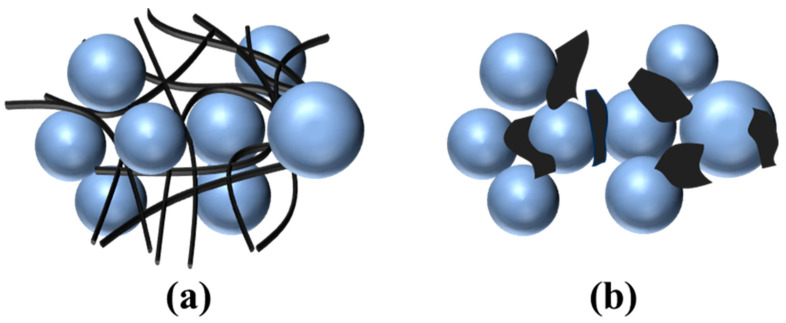
(**a**) Fabrication of an interfacial heat conduction network between filler particles by adding CNTs. (**b**) Dispersion of GNSs among the filler particles.

The proposed mechanism for CNTs to enhance the λ values of EMCs is illustrated in Figure 6a. When incorporated into ECMs, CNTs, which possess excellent intrinsic TC, can create effective heat conduction pathways within the matrix and facilitate more efficient heat dissipation. Moreover, as the CNT concentration increases to a sufficient threshold, CNTs begin to bridge together, forming an interconnected thermal network. This network enables more efficient heat transfer along the CNT axis while reducing heat scattering. In addition, due to their nanoscale size and curled structure, CNTs can also reduce the ITR by forming strong interactions with fillers and polymers and provide more efficient heat transfer. Therefore, the high intrinsic TC, the formation of a heat conduction network, and the reduced ITR are three primary factors enhancing the TC of EMCs when using CNTs.

In contrast, GNSs exhibit orientation-dependent heat transfer, the lack of a continuous heat conduction network, and high ITR, making them less effective than CNTs for improving the TC of EMCs. Due to their planar structure, GNSs possess high in-plane TC but low cross-plane TC. When dispersed in a polymer matrix, GNSs are typically flat and randomly oriented (Figure 6b), which restricts heat transfer perpendicular to the CNS plane. Furthermore, their random dispersion fails to establish a well-connected thermal conduction network, while their tendency to aggregate or stack can even act as a thermal barrier to impede heat transfer. Additionally, the planar structure of GNSs results in weak interfacial adhesion with fillers, leading to relatively high ITR at these interfaces. Collectively, these factors render GNSs ineffective or even detrimental to the TC of EMCs.

### 3.4. Topographic Characterization of Filler Surfaces

To further investigate the structural characteristics of different fillers, the surface morphologies of different fillers and additives were examined using FESEM, and the results are illustrated in Figure 6.

**Figure 7:**
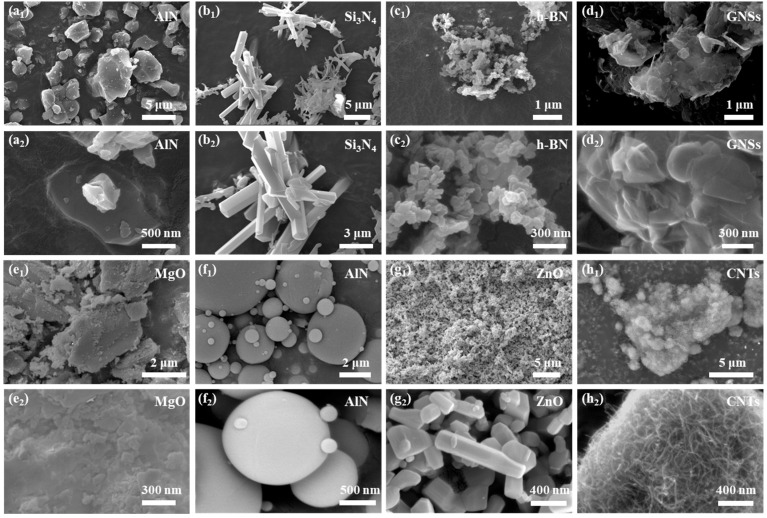
SEM micrographs of various fillers at different magnifications: (**a_1_**,**a_2_**) AlN. (**b_1_**,**b_2_**) Si_3_N_4_. (**c_1_**,**c_2_**) h-BN. (**d_1_**,**d_2_**) GNSs. (**e_1_**,**e_2_**) MgO. (**f_1_**,**f_2_**) Al_2_O_3_. (**g_1_**,**g_2_**) ZnO. (**h_1_**,**h_2_**) CNTs.

As shown in Figure 7a_1_,a_2_, AlN fillers exhibit a granular morphology with dispersed distribution. The Si_3_N_4_ fillers (Figure 7b_1_,b_2_) appear as hexagonal prism-shaped particles that form clusters. h-BN consists of small and densely packed particles (Figure 7c_1_,c_2_). SEM images of GNSs (Figure 7d_1_,d_2_) reveal a flake-like morphology with obvious agglomeration. MgO particles have a small particle size and tend to cluster together at higher magnifications (Figure 7e_1_,e_2_). The surface morphology of commercial Al_2_O_3_ filler particles (Figure 7f_1_,f_2_) consists of spherical particles with different particle sizes. The morphology of ZnO particles is similar to that of silicon nitride, which is also distributed in hexagonal prisms (Figure 7g_1_,g_2_), and its particle size is in the nanometer scale. The carbon nanotube filler exhibited a characteristic tubular structure, as shown in Figure 7h_1_,h_2_. The morphology of fillers is a key factor in regulating the properties of composite materials, directly affecting the construction of thermal conductivity networks and the feasibility of processes.

### 3.5. Surface Morphology and Elemental Distribution of EMCs

**Figure 8:**
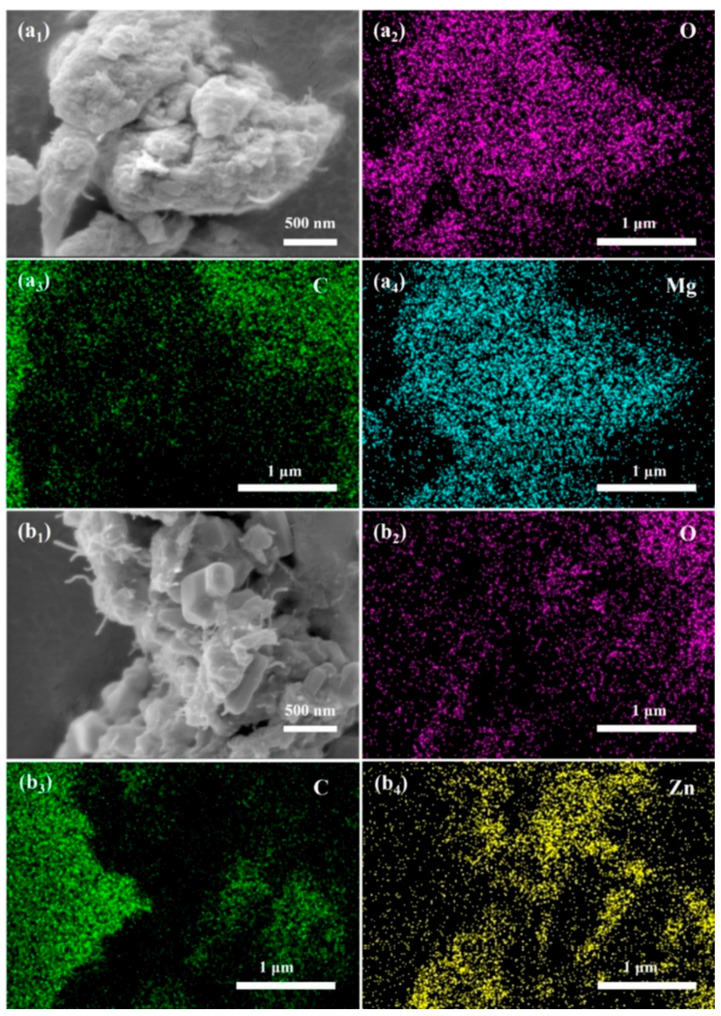
SEM images and energy dispersion spectra (EDS) of EMCs with 60 wt.% MgO + 2 wt.% CNTs and 60 wt.% ZnO + 2 wt.% CNTs fillers: (**a_1_**–**a_4_**) 60 wt.% MgO + 2 wt.% CNTs; (**b_1_**–**b_4_**) 60 wt.% ZnO + 2 wt/% CNTs.

To elucidate the role of CNTs in improving the λ values of the prepared EMCs, surface morphology observation and surface elemental scanning analysis were performed using SEM after blending six inorganic fillers with CNTs in a twin-screw extruder. The images of EMCs containing 60 wt.% MgO + 2 wt.% CNTs and 60 wt.% ZnO + 2 wt.% CNTs were selected as representative examples for further discussion, while images for the other samples are shown in Figure S1 in the Supplementary Materials. The surface morphology of the composite with 60 wt.% MgO + 2 wt.% CNTs is depicted in Figure 8a_1_, showing that the gaps between the MgO particles were filled with a small amount of CNTs, and extensive CNT coverage was observed on the filler surface. The elemental analysis results (Figure 8a_2_–a_4_) showed a uniform distribution of all three elements—magnesium, oxygen, and carbon—indicating the homogeneous dispersion of CNTs within the inorganic filler.

Similarly, the surface morphology of 60 wt.% ZnO + 2 wt.% CNTs composites (Figure 8b_1_) revealed CNTs filling the gaps between hexagonal ZnO particles, with the elemental analysis (Figure 8b_2_–b_4_) confirming the uniform distribution of CNTs. These results suggest that CNTs effectively fill the voids between the inorganic fillers, replacing air with highly thermally conductive CNTs, thereby forming an efficient heat conduction network throughout the filler material.

### 3.6. FT-IR of EMCs

**Figure 9:**
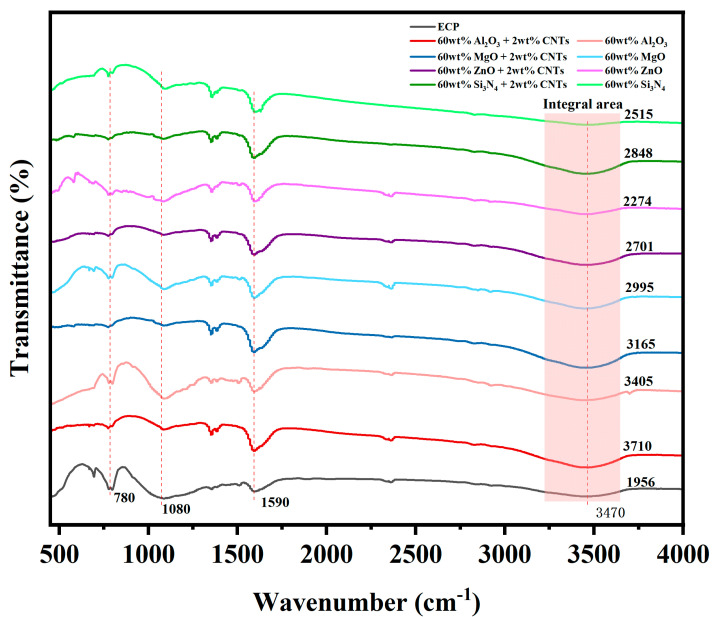
FT-IR spectra of EMCs containing different fillers.

The characteristic peak at 780 cm^−1^ represents the C-H out-of-plane bending vibration of the benzene ring in the phenolic resin matrix [41,42,43]. The distinctive peak at 1080 cm^−1^ can be attributed to the C-O stretching vibration of the secondary alcohols formed during epoxy curing [43,44], and the broad peak absorption at 3470 cm^−1^ can be assigned to the stretching vibration of the –OH group [43,44]. As shown in Figure 9, the intensities of the residual epoxy-related peaks in EMCs containing CNTs were significantly reduced, while the intensity of the –OH peaks of the hydroxyl groups (formed via epoxy ring-opening and curing) increased. To quantitatively validate this observation, the broad –OH absorption band within 3220–3650 cm^−1^ was subjected to baseline correction and Gaussian peak deconvolution (annotated in Figure 9). The integrated area of the –OH peak demonstrated a marked enhancement upon CNT addition: for instance, the EMC with 60 wt.% Al_2_O_3_ filler showed an increase from 3405 (without CNTs) to 3710 (with 2 wt.% CNTs), indicating accelerated crosslinking kinetics. These results confirm that CNTs promote the curing reaction between epoxy resin and the curing agent by facilitating epoxy ring-opening and hydroxyl group formation, thereby enhancing interfacial bonding and reducing thermal resistance.

FT-IR analysis shows that carbon nanotubes enhance the curing kinetics and interfacial bonding of epoxy resin through physical and chemical interactions: (1) weakened residual epoxy resin-related peaks (e.g., 780 cm^−1^) and enhanced –OH signals (3470 cm^−1^) imply that carbon nanotube-induced physical adsorption/hydrogen bonding accelerates the ring opening and crosslinking of epoxy groups; (2) stable C=C stretching vibrations (1590 cm^−1^) validated the complete aromatic structure, indicating non-destructive carbon nanotube resin binding [45]. These mechanisms synergistically reduced the interfacial thermal resistance, thereby increasing the thermal conductivity (1.14 W/m · K, Table S1). In summary, CNTs provide physicochemical mechanism support for improving thermal conductivity (λ = 1.14 W/m·K) by optimizing curing kinetics and interface bonding to reduce interfacial thermal resistance (ITR).

### 3.7. Thermal Conductive Testing of EMCs

**Figure 10:**
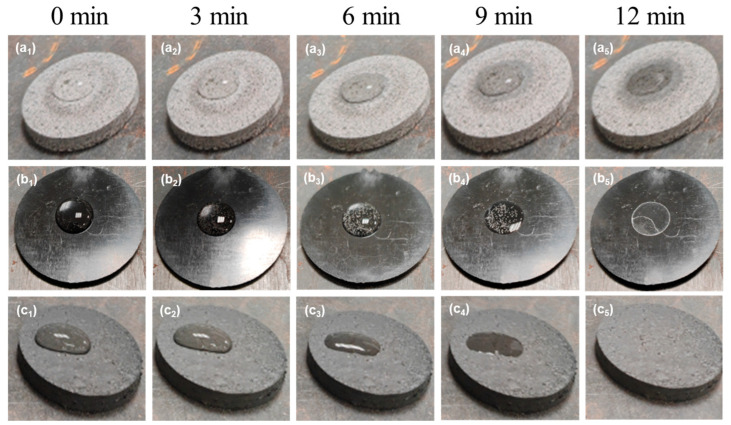
Experimental image of water evaporation at 150 °C. (**a_1_**–**a_5_**) ECP. (**b_1_**–**b_5_**) EMC with 90 wt.% Al_2_O_3_. (**c_1_**–**c_5_**) EMC with 60 wt.% Si_3_N_4_ + 2wt.% CNTs.

To further validate the improvement in λ values of the composite materials, a water evaporation experiment was conducted using three materials, i.e., ECP, EMC containing 90 wt.% Al_2_O_3_, and EMC containing 60 wt.% Si_3_N_4_+2 wt.% CNTs. The thermal conductivity of the composites was indirectly evaluated by measuring the evaporation rates of identical water droplets on various EMC surfaces under isothermal conditions, with comparative experimental results as systematically summarized in Figure 10.

As illustrated in Figure 10, no significant reduction in the volume of water droplets was observed on the ECP material during the initial 12-minute test (Figure 10a_1_–a_4_). In contrast, the water droplets on EMC with 90 wt.% Al_2_O_3_ exhibited a notable decrease in volume, with complete evaporation occurring between 9 min and 12 min, as depicted in Figure 10b_4_,b_5_. Additionally, a significant reduction in water droplet volume was observed between 6 min and 9 min (Figure 9b_3_,b_4_). As illustrated in Figure 10c_2_,c_3_, the EMC material with 60 wt.% Si_3_N_4_ + 2 wt.% CNTs exhibited a marked increase in water droplet evaporation between 3 min and 6 min. A comparison of the prepared EMCs containing fillers and CNTs with the EMCs containing only fillers revealed that the former exhibited a faster water evaporation rate (Figure 10c_2_,c_3_,b_2_–b_3_), indicating the enhancement of TC by adding CNTs as additives. Thus, the incorporation of CNTs has successfully established efficient heat conduction channels within the polymer matrix and enhanced the λ values of the EMCs.

## 4. Conclusions

In summary, to improve the heat conduction performance of EP packaging materials, six inorganic filler materials were chosen and were incorporated into the ECP by a twin-screw extruder. The λ values of the resulting EMCs were enhanced to 0.4 W/m∙K compared to the pristine ECP. However, it was found that interfacial thermal resistance between the fillers and EP significantly restricted the efficiency of heat conduction improvement. To solve this issue, we propose that by adding CNTs into the EP composite, one could reduce the filler content and improve the λ values. The prepared EMCs with CNTs improved the λ values of ECP by more than 216%. The optimized λ values of EMC reached 1.14 W m^−1^∙K^−1^ by mixing 60 wt.% Si_3_N_4_ with 2 wt.% CNTs in ECP, which is comparable to that of EMC with 90 wt.% Al_2_O_3_. It was proposed that CNTs can effectively fill the voids between fillers and EP, thus helping to establish an effective heat-conductive network within the EP matrix. This result suggests the presence of a synergistic effect between CNTs and inorganic fillers, which may contribute to the enhancement of thermal properties in EMCs. Future studies will optimize the rheology of CNT/Si_3_N_4_ (60 wt.%) systems by tailoring the interfaces and processing to reduce viscosity, while retaining thermal conductivity and volumetric resistivity (as detailed in Supplementary Tables S4 and S5), enabling the scalable preparation of high-density electronic packaging materials.

## Figures and Tables

**Table 1:** Information about the chemical reagents used in experiments.

Reagent Name	Manufacturer	Purity
Linear phenolic resin	Shandong Shengquan New Material Co., Ltd. (Jinan, China)	CP
Triphenylphosphine	Shandong Shengquan New Material Co., Ltd. (Jinan, China)	CP
Fused silica micro powder	Shandong Shengquan New Material Co., Ltd. (Jinan, China)	AR
*o*-cresol epoxy resin	Sinopec Baling Petrochemical Co., Ltd. (Yueyang, China)	CP
Carbon nanosheets (GNSs)	Anhui Zesheng Technology Co., Ltd. (Anqing, China)	CP
Carbon nanotubes (CNTs)	Tianjin Hiens Optronics Co., Ltd. (Tianjin, China)	CP
Aluminum nitride (AlN)	Dongguan Zhan Yang Materials Co., Ltd. (Dongguan, China)	CP
Hexagonal boron nitride (h-BN)	Anhui Senrise Technology Co., Ltd. (Anqing, China)	CP
Magnesium oxide (MgO)	Chengdu Huaxia Chemical Reagent Co., Ltd. (Chengdu, China)	CP
Aluminum oxide (Al_2_O_3_)	Jiangsu Lianrui New Materials Co., Ltd. (Lianyungang, China)	CP
Zinc oxide (ZnO)	Fushi New Material Technology Co., Ltd. (Tianjin, China)	CP
Silicon nitride (Si_3_N_4_)	Chengdu Dianchun Technology Co., Ltd. (Chengdu, China)	CP

Note: CP = chemically pure; AR = analytical research grade.

**Table 2:** Formulation and composition of the prepared ECP.

Materials	Quality/g
Phenolic resin	100
*o*-cresol Epoxy resin	200
Triphenylphosphine	0.9
Fused silica micro powder	700

**Table 3:** λ values of the various fillers in their bulk crystal state.

Filler	Theoretical λ Values (W m^−1^∙K^−1^)	Reference
Al_2_O_3_	10–30	[26]
MgO	40	[27]
ZnO	30	[28]
Si_3_N_4_	180	[29]
h-BN	250–380	[30]
AlN	320	[31]

**Table 4:** λ value of EMCs incorporating different inorganic fillers.

Entry	Filler	Filler Contents	λ Values (W m^−1^ K^−1^)
1	N/A	N/A	0.36
2	Al_2_O_3_	90 wt.%	1.19
3	AlN	90 wt.%	0.82
4	Si_3_N_4_	90 wt.%	0.67
5	Al_2_O_3_	60 wt.%	0.67
6	AlN	60 wt.%	0.44
7	Si_3_N_4_	60 wt.%	0.86
8	MgO	60 wt.%	0.49
9	ZnO	60 wt.%	0.76
10	h-BN	60 wt.%	0.82

## Data Availability

The data presented in this study are available on request from the corresponding author. The data are not publicly available due to privacy.

## Supplementary Materials

The following supporting information can be downloaded at: https://www.mdpi.com/article/10.3390/polym17111507/s1. Figure S1: SEM images and energy spectra of four epoxy molding compounds with different fillers; Figure S2: SEM images of EMCs with ZnO and MgO; Table S1: λ values of phenolic epoxy electronic packaging materials after adding carbon nanotubes and graphene nanosheets; Table S2: Comparison of the thermal conductivity performance of the prepared packaging materials with previously published results [13,46,47,48,49,50,51,52,53]; Table S3: Thermal conductivity measurements of six inorganic filler powders; Table S4: Volume resistivity of ECP material; Table S5: Volume resistivity of EMC material (60 wt.% Si3N4 + 2 wt.% CNTs).
