# Supplementary material for: Preparation of Phenolic Epoxy-Based Electronic Packaging Materials with High Thermal Conductivity by Creating an Interfacial Heat Conduction Network
Source: Polymers (Basel). 2025 May 28;17(11):1507. doi: 10.3390/polym17111507 (PMC12158111; doi:10.3390/polym17111507)
Supplement: Supplementary file 1 [file polymers-17-01507-s001.zip › polymers-3624278-supplementary.pdf]

Supporting Information

Synthesis of High Thermal Conductivity Phenolic Epoxy Electronic Packaging Materials by Creating Interfacial Heat Conduction Network

Minghao Ye[1], Jing Jiang[1], Lin Zhao[1], Hongyu Zhu[1], Junjie Wang*[, 2], Zicai Sun*[, 3], Dewei Zhang[4], Ming Li[4] and Yagang Zhang*[, 1]

[1] School of Materials and Energy, University of Electronic Science and Technology of China, Chengdu 611731, Sichuan, China
[2] Department of Chemistry, School of Science, Xihua University, Chengdu 610039, Sichuan, China
[3] Dongguan Yimei Material Technology Co., Ltd., Dongguan, 523000, Guangdong, China
[4] Sichuan Xinyi Electronic Materials Co., Ltd., Chengdu 611731, Sichuan, China
* Correspondence: ygzhang@uestc.edu.cn (Y.Z.); jjwang@xhu.edu.cn (J.W.); sunzic@mails.ucas.ac.cn (Z.S.)

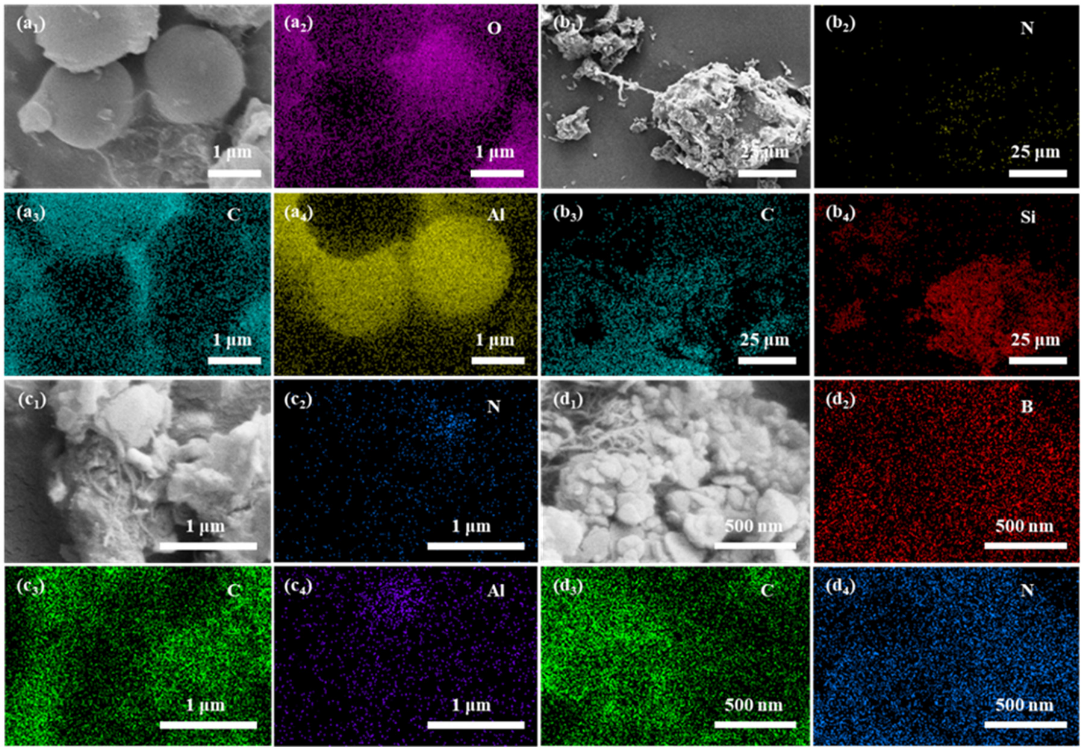

Figure S1 SEM images and energy spectra of four epoxy molding compounds with different fillers. (a1) 60 wt% Al_2O_3 + 2 wt% CNTs. (b) 60 wt% Si_3N_4 + 2 wt% CNTs. (c) 60 wt% AlN + 2 wt% CNTs. (d) 60 wt% h-BN + 2 wt% CNTs..

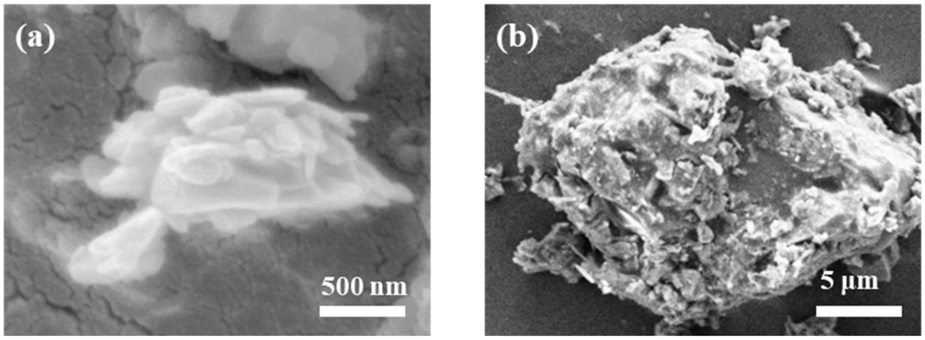

Figure S2 SEM images of EMCs with ZnO and MgO. (a) EMCs with 60 wt% ZnO. (b) EMCs with 60 wt% MgO.

Table S1. λ values of phenolic epoxy electronic packaging materials after adding carbon nanotubes and graphene nanosheets.

Number	Type of filler	Power (W)	Fill volume	TC (W/m·K)
#1	Al_2O_3 + CNTs	0.50	60 wt%+2 wt%	0.88
#2	Al_2O_3 + GNSs	0.50	60 wt%+2 wt%	0.62
#3	ZnO + CNTs	0.40	60 wt%+2 wt%	0.95
#4	ZnO + GNSs	0.40	60 wt%+2 wt%	0.65
#5	Si_3N_4+ CNTs	0.60	60 wt%+2 wt%	1.14
#6	Si_3N_4+ GNSs	0.60	60 wt%+2 wt%	0.91
#7	MgO + CNTs	0.48	60 wt%+2 wt%	0.68
#8	MgO + GNSs	0.48	60 wt%+2 wt%	0.64
#9	h-BN + CNTs	0.50	60 wt%+2 wt%	1.10
#10	h-BN + GNSs	0.50	60 wt%+2 wt%	0.83
#11	AlN + CNTs	0.50	60 wt%+2 wt%	0.74
#12	AlN + GNSs	0.50	60 wt%+2 wt%	0.42

Table S2. Comparison of thermal conductivity performance of the prepared packaging materials with previously published work.

Type of resin	Type of filler	Fill volume	λ values (W/m·K)	Ref.
Bisphenol A epoxy resin	h-BN	15 vol%	0.70	[46]
Bisphenol F epoxy resin	Al_2O_3/BN	80 wt%	1.72	[13]
Bisphenol A epoxy resin	BN	60 wt%	1.05	[47]
Bisphenol type epoxy resin	GNPs	2.70 vol%	0.75	[48]
Commercial epoxy resins	GNPs	5 wt%	0.48	[49]
Bisphenol A diglycidyl	BN/SiO_2	50 wt%	0.73	[50]
EPON 128	MCNTs	5 vol%	0.96	[51]
Glycidyl methacrylate	Graphene	4 phr	1.91	[52]
Bisphenol A epoxy	h-BN	30 wt%	1.18	[53]
O-cresol epoxy resin	Si_3N_4+CNTs	60 wt%+2 wt%	1.14	This work

Table S3. Thermal conductivity measurements of six inorganic filler powders.

Sample	Test power (W)	Thermal conductivity (W/m·K)
ZnO	0.18	0.16
MgO	0.18	0.12
AlN	0.15	0.15
Si_3N_4	0.18	0.23
h-BN	0.15	0.18
Al_2O_3	0.25	0.51

Table S4. Volume resistivity of ECP material.

Location number	Thickness (cm)	Cross-sectional area (cm^2)	Volume resistance (TΩ)	Volume resistivity (TΩ/cm)	Electrical conductivity (pS/cm)
**A**	0.3	19.63	35.2	2303	0.00043417
**B**	0.3	19.63	28.6	1871	0.00053436
**C**	0.3	19.63	24.1	1577	0.00063414

$T=10^{12}$，$G=10^9$, $M=10^6$, $k=10^3$， $m=10^{-3}$，$\mu=10^{-6}$，$n=10^{-9}$, $p=10^{-12}$

Table S5. Volume resistivity of EMC material (60 wt% Si_3N_4 + 2 wt% CNTs).

Location number	Thickness (cm)	Cross-sectional area (cm^2)	Volume resistance (MΩ)	Volume resistivity (MΩ/cm)	Electrical conductivity (μS/cm)
**A**	0.47	12.5	3.53	9.39	0.001065156
**B**	0.47	12.5	4.19	11.1	0.000897375
**C**	0.47	12.5	3.11	8.27	0.001209003

$T=10^{12}$，$G=10^9$, $M=10^6$, $k=10^3$， $m=10^{-3}$，$\mu=10^{-6}$，$n=10^{-9}$, $p=10^{-12}$

References:

46. M. Liu, H. Zhang, Y. Wu, D. Wang, L. Pan, Effect of functionalization on thermal conductivity of hexagonal boron nitride/epoxy composites, International Journal of Heat and Mass Transfer 219 (2024) 124844.

47. J. Gu, Q. Zhang, J. Dang, C. Xie, Thermal conductivity epoxy resin composites filled with boron nitride, Polymers for Advanced Technologies 23(6) (2012) 1025-1028.

48. C. Min, D. Yu, J. Cao, G. Wang, L. Feng, A graphite nanoplatelet/epoxy composite with high dielectric constant and high thermal conductivity, Carbon 55 (2013) 116-125.

49. F. Wang, L.T. Drzal, Y. Qin, Z. Huang, Mechanical properties and thermal conductivity of graphene nanoplatelet/epoxy composites, Journal of Materials Science 50(3) (2015) 1082-1093.

50. Y.-H. Song, L.-J. Yin, S.-L. Zhong, Q.-K. Feng, H. Wang, P. Zhang, H.-P. Xu, T. Liang, Z.-M. Dang, A processable high thermal conductivity epoxy composites with multi-scale particles for high-

frequency electrical insulation, Advanced Composites and Hybrid Materials 7(4) (2024) 115.

51. S.-Y. Yang, C.-C.M. Ma, C.-C. Teng, Y.-W. Huang, S.-H. Liao, Y.-L. Huang, H.-W. Tien, T.-M. Lee, K.-C. Chiou, Effect of functionalized carbon nanotubes on the thermal conductivity of epoxy composites, Carbon 48(3) (2010) 592-603.
52. C.-C. Teng, C.-C.M. Ma, C.-H. Lu, S.-Y. Yang, S.-H. Lee, M.-C. Hsiao, M.-Y. Yen, K.-C. Chiou, T.-M. Lee, Thermal conductivity and structure of non-covalent functionalized graphene/epoxy composites, Carbon 49(15) (2011) 5107-5116.
53. J. Hou, G. Li, N. Yang, L. Qin, M.E. Grami, Q. Zhang, N. Wang, X. Qu, Preparation and characterization of surface modified boron nitride epoxy composites with enhanced thermal conductivity, Rsc Advances 4(83) (2014) 44282-44290.
